# UV-Engineered Oxygen Vacancies in MoO_X_ Interlayers Enable 24.15% Efficiency for Crystalline Silicon Solar Cells

**DOI:** 10.3390/ma18225167

**Published:** 2025-11-13

**Authors:** Linfeng Yang, Wanyu Lu, Jingjie Li, Shaopeng Chen, Tinghao Liu, Dayong Yuan, Yin Wang, Ji Zhu, Hui Yan, Yongzhe Zhang, Qian Kang

**Affiliations:** 1 Key Laboratory Optoelectronics Technology, Ministry of Education, School of Information Science and Technology, Beijing University of Technology, Beijing 100124, China; linfengy2025@163.com (L.Y.); 15694899873@163.com (W.L.); chenshaopeng@emails.bjut.edu.cn (S.C.); dayongyuan@emails.bjut.edu.cn (D.Y.); wangyin_michaelb@163.com (Y.W.); zjfromdark@outlook.com (J.Z.); yzzhang@bjut.edu.cn (Y.Z.); 2 School of Physics and Optoelectronic Engineering, Beijing University of Technology, Beijing 100124, China; ljj1992@bjut.edu.cn; 3 School of Materials Science and Engineering, Beijing University of Technology, Beijing 100124, China; liutinghao010206@163.com (T.L.); hyan@bjut.edu.cn (H.Y.)

**Keywords:** oxygen vacancy, ultraviolet irradiation, MoO_X_, hole transport layer, crystalline silicon solar cell

## Abstract

Molybdenum oxide (MoO_X_) has been widely utilized as a hole transport layer (HTL) in crystalline silicon (*c*-Si) solar cells, owing to characteristics such as a wide bandgap and high work function. However, the relatively low conductivity of MoO_X_ films and their poor contact performance at the MoO_X_-based hole-selective contact severely degrade device performance, particularly because they limit the fill factor (FF). Oxygen vacancies are of paramount importance in governing the conductivity of MoO_X_ films. In this work, MoO_X_ films were modified through ultraviolet irradiation (UV-MoO_X_), resulting in MoO_X_ films with tunable oxygen vacancies. Compared to untreated MoO_X_ films, UV-MoO_X_ films contain a higher density of oxygen vacancies, leading to an enhancement in conductivity (2.124 × 10^−3^ S/m). In addition, the UV-MoO_X_ rear contact exhibits excellent contact performance, with a contact resistance of 20.61 mΩ·cm^2^, which is significantly lower than that of the untreated device. Consequently, the application of UV-MoO_X_ enables outstanding hole selectivity. The power conversion efficiency (PCE) of the solar cell with an n-Si/i-a-Si:H/UV-MoO_X_/Ag rear contact reaches 24.15%, with an excellent FF of 84.82%.

## 1. Introduction

Crystalline silicon (*c*-Si) solar cells continue to dominate the photovoltaic market, primarily owing to their abundant raw material resources, extended operational lifetime, and high power conversion efficiency (PCE) [1,2,3]. Silicon heterojunction (SHJ) cells attain superior passivation performance by employing double-sided i-a-Si:H passivation layers. Meanwhile, the doped silicon layers, namely n-type amorphous silicon (n-a-Si:H) and p-type nanocrystalline silicon (p-nc-Si:H), demonstrate excellent carrier selectivity, ultimately leading to a high PCE of 26.81% [4]. Notably, integrating heterojunction with back-contact (IBC) structures has further pushed certified cell efficiencies of 27.81% [5]. However, the utilization of doped silicon films in SHJ solar cells poses several challenges. Firstly, the fabrication of doped silicon films involves costly deposition processes and the use of toxic gas precursors [6]. Moreover, their narrow bandgap (*E*_g_) leads to optical parasitic absorption and free carrier absorption losses, which can constrain the enhancement of the short circuit current density (*J*_sc_) in solar cells [7,8,9]. To address these issues of parasitic absorption and process cost, researchers have used wide-bandgap materials instead of doped silicon layers, such as transition metal oxides (TMOs), alkali metal fluorides [10,11,12,13,14], and organic compounds [15,16,17]. Among these materials, easily fabricable TMOs with a high work function—such as MoO_X_ [18,19], V_2_O_X_ [20], WO_X_ [21,22,23] and CrO_X_ [24]—are considered to be promising hole transport layers (HTLs) and have attracted extensive research interest. High-W_F_ TMOs in non-stoichiometric forms contain oxygen vacancies. The density of oxygen vacancies is closely related to the film’s properties, including work function and conductivity. As the valence state of the metal cation in TMOs decreases, attributed to an increase in oxygen vacancy density, the W_F_ of these TMOs decreases [25]. On the other hand, the conductivity of high-W_F_ TMOs originates from oxygen vacancies that donate electrons, resulting in n-type conductivity [26,27]. Among various TMO materials, MoO_X_ demonstrates superior performance due to its higher work function and wider bandgap. Upon the deposition of MoO_X_ via thermal evaporation for use as the HTL, the solar cell attains the highest device efficiency, at 23.83%; yet, it simultaneously exhibits a high contact resistance (*ρ*_c_) of 177 mΩ·cm^2^ [28]. In addition, MoO_X_ exhibits poor film conductivity [29]. The high *ρ*_c_ and low conductivity limit the transport of photogenerated holes, thereby limiting the improvement of fill factor (FF) and *J*_sc_ [30,31]. As predicted by Gao et al., an excellent FF (>83%) can be achieved when *ρ*_c_ is below 80 mΩ·cm^2^ and contact recombination current density (*J*_0c_) is less than 5 fA·cm^−2^ [32].

Enhancing the electrical performance of MoO_X_-based *c*-Si compound cells, including reducing contact resistance and improving conductivity, is an essential approach for achieving high-efficiency cells. Li et al. used MoO_2_ as the evaporation source to fabricate MoO_X_ films with a low oxygen content. Compared to high-oxygen-content films, these low-oxygen-content films demonstrated superior contact performance, with *ρ*_c_ decreasing from 121.38 mΩ·cm^2^ to 15.06 mΩ·cm^2^ [33]. However, when MoO_2_ serves as an evaporation precursor, it undergoes disproportionation during the high-temperature, oxygen-free process, leading to the formation of products like MoO_3_ and consequently complicating the control of film composition [34]. Research has revealed that adopting a bilayer structure can effectively enhance the conductivity of the film. Li et al. found that a MoO_X_/NiO_X_ bilayer HTL structure exhibits higher conductivity compared to a single layer (MoO_X_ or NiO_X_). Moreover, the MoO_X_/NiO_X_ bilayer optimizes the energy band structure, thereby promoting hole-elective transport and resulting in a high FF [35]. In a separate study by Lu et al., a MoO_X_/Au/MoO_X_ (MAM) multilayer composite used as the HTL, effectively improved the device’s contact performance, as reflected by a lower *ρ*_c_ value of 62.42 mΩ·cm^2^. The incorporation of a Au interlayer promotes the generation of oxygen vacancies in MoO_X_, thereby increasing the conductivity of the MAM composite HTL, which ranges from 8.02 × 10^−3^ S/m to 7.592 × 10^−2^ S/m, facilitating hole extraction, and achieving a high FF of 85.2% [36]. However, introducing Au into the MoO_X_/Au/MoO_X_ multilayer raises device costs. Although multilayer HTLs demonstrate significant promise for enhancing film conductivity and contact performance, the fabrication of bilayer or multilayer structures inevitably adds to the complexity of the manufacturing process.

In this work, thermally evaporated MoO_X_ was employed as the HTL for n-Si solar cells, and tunable oxygen vacancy density in MoO_X_ films was achieved via ultraviolet (UV) irradiation for varying durations. X-ray photoelectron spectroscopy (XPS) analysis revealed that as UV exposure time increased, both the Mo^6+^/Mo^5+^ ratio and O/Mo ratio gradually decreased. This trend clearly indicates that UV irradiation induces a reduction in MoO_X_, leading to a corresponding increase in oxygen vacancy density. By comparing the MoO_X_ film with optimal UV exposure of 60 min, denoted as UV-MoO_X_, to the untreated MoO_X_ film, the UV-MoO_X_ film exhibited a higher conductivity of 2.124 × 10^−3^ S/m. Meanwhile, ultraviolet photo-electron spectroscopy (UPS) measurements showed that the W_F_ of the UV-MoO_X_ film was 5.05 eV, which is nearly comparable to that of the untreated MoO_X_ film (W_F_ = 5.10 eV). In essence, a MoO_X_ film with both high conductivity and a high W_F_ was achieved via 60 min of UV irradiation. Contact resistance measurements further confirmed superior contact performance when using the UV-MoO_X_ film as HTL, with the *ρ*_c_ decreasing from 91.21 mΩ·cm^2^ to 20.61 mΩ·cm^2^. Moreover, by comparing the experimental current–voltage (*I*–*V*) curve and the pseudo-*I*–*V* curve derived from Suns-*V*_oc_ measurements, we found that the device with UV-MoO_X_ as the HTL exhibited a lower series resistance of 0.434 Ω·cm^2^ and a higher pseudo-fill factor (pFF) of 86.84%. This UV treatment strategy effectively enhanced the device’s electrical performance, resulting in an FF of 84.82% and an open circuit voltage (*V*_oc_) of 729 mV. The successful application of UV-MoO_X_ in *c*-Si solar cells provides valuable new insights into the controllable modulation of properties in high-W_F_ TMO materials.

## 2. Materials and Methods

### 2.1. Device Fabrication

n-type silicon compound heterojunction solar cells were fabricated using n-Si wafers with a resistivity and thickness of 1.2–1.5 Ω·cm and 130 μm, respectively. The n-Si wafers were first cleaned and textured via a wet chemical process. An 8 nm i-a-Si:H thin film was prepared using radio frequency plasma-enhanced chemical vapor deposition (PECVD) on the front and back of the silicon wafer. After that, an n-nc-SiOx:H layer was deposited on the front and a SiN_X_ layer on the back, using PECVD. On the front side of the cell, the TCO layer was deposited using magnetron sputtering, consisting of In_2_O_3_ doped with 10 wt% SnO_2_. The silver grid electrode was printed and then annealed at 190 °C for 30 min. Before depositing MoO_X_, the rear side was cleaned using HF at a concentration of 2%. MoO_X_ (approximately 7 nm) and Ag (200 nm) were thermally evaporated onto the rear of the solar cell at rates of 0.1 Å/s and 1.5 Å/s, respectively. This structure served as the control group. Additionally, for the samples treated with ultraviolet lamp, an approximately 7 nm MoO_X_ film was deposited on the rear of the solar cell via thermal evaporation, at a rate of 0.1 Å/s. The chamber was then opened, and the MoO_X_ film was exposed to ultraviolet lamps (wavelength 365 nm, power 7 W) for various times (30 min, 60 min, and 120 min) in a glove box. The UV spot was configured as a circular area with a radius of 10 cm, while the sample size was 4 × 4 cm^2^. Since the spot size was larger than the sample, the entire device surface received uniform UV irradiation during treatment. Following this, a 200 nm Ag film was deposited using thermal evaporation at a rate of 1.5 Å/s. Throughout the thermal evaporation process, the pressure inside the chamber remained below 5 × 10^−4^ Pa. Both the thermal evaporation and ultraviolet treatment processes were conducted in a nitrogen environment. The brief vacuum interruption between the MoO_X_ and Ag deposition did not adversely affect device performance. For detailed experimental data, please refer to Table S1. The detailed experimental flowchart is provided in Figure S1 of the Supporting Information.

### 2.2. Material Characterization

The work function, elemental composition, and chemical state of the film were characterized by UPS and XPS. These analyses utilized a monochromatic Al Kα X-ray source with a photon energy of 1486.7 eV, implemented on a ESCALab 250Xi measurement system (Thermo Fisher Scientific, Waltham, MA, USA). All acquired XPS spectra were processed and deconvoluted using Advantage 5.99 software, employing a mixed Lorentz–Gaussian (GL) line shape for fitting. Additionally, all spectra were calibrated using the carbon C1s peak at 284.8 eV as a reference. The film conductivity was measured by the two-probe method using a 2450 digital source meter (Keithley, Beaverton, OR, USA) and computationally fitted. The test samples were composed of p-Si/Ag/MoO_X_/Ag and p-Si/Ag/UV-MoO_X_/Ag structures. A 200 nm layer of Ag was initially deposited onto a p-Si wafer. Subsequently, a mask with circular holes of different diameters was applied to define the area within which either MoO_X_ or UV-MoO_X_ was deposited. This was followed by another 200 nm of Ag through thermal evaporation deposition. The *ρ*_c_ measurements were performed using the ECSM. More details on the calculation methods are provided in Note S1, Supporting Information.

### 2.3. Solar Cell

The light and dark *J*–*V* characteristics of the solar cell (4 × 4 cm^2^) were recorded using a Class AAA Scisun solar simulator (Sciencetech, London, ON, Canada) and a Keithley 2450 source meter under standard 1 sun conditions (100 mW·cm^−2^, AM 1.5 G spectra, 25 °C), and the luminescence intensity was calibrated using a certified Fraunhofer Cal Lab reference cell. The EQE was obtained using a QE-R (Enlitech, Kaohsiung City, Taiwan) measurement system. The dark *τ*_eff_ and *J*_0c_ values were evaluated using the QSSPC method. The device architectures used in the test were MoO_X_/i-a-Si:H/n-Si/i-a-Si:H/MoO_X_ and UV-MoO_X_/i-a-Si:H/n-Si/i-a-Si:H/UV-MoO_X_, enabling the measurements of *τ*_eff_ with a lifetime tester (WCT-120MX) (Sinton Instruments, Boulder, CO, USA). The pseudo-light-*J*–*V* curves were extracted from a Suns-*V*_oc_ measurement. The Suns-*V*_oc_ module of a Sinton WCT-120 instrument (Sinton Instruments, Boulder, CO, USA) was used to collect changes in the voltage of the device by reducing the light intensity of the flashlight; these were computationally transformed into Suns-*V*_oc_ curves.

### 2.4. Simulation

Quokka2 was utilized for the PLA of the solar cells with MoO_X_ and UV-MoO_X_. For the Quokka2 simulation, the unit cell was modeled in two dimensions to calculate the power loss in the transversal transport of carriers. The input parameters were primarily obtained from the measurements described in Section 2.2. The line resistance of the finger and the contact resistivity of the heterojunction were considered to be series resistance in an external circuit. The optical path-length factor (Z) was set as 4n^2^: the simulated *J*_sc_ to that of actual cells. Richter’s Auger mode was chosen, and the value of radiation recombination was changed to 0.4 × Brad, with a photon recycling probability of 0.6. Other parameters employed in the simulation are listed in Table S2.

## 3. Results and Discussion

**Figure 1:**
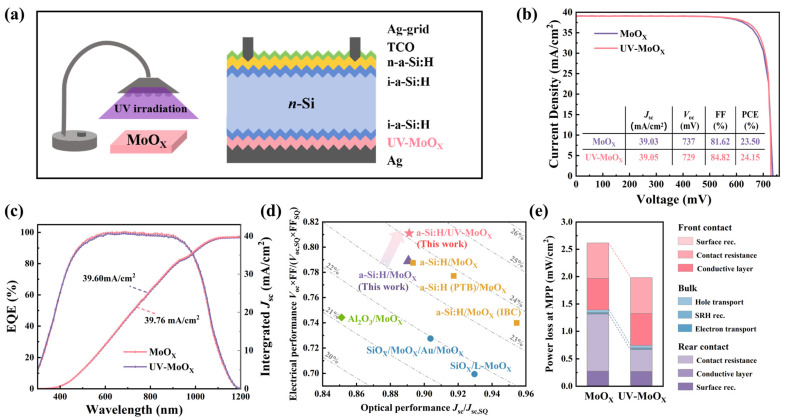
(**a**) The process of UV treatment MoO_X_ and the cross-sectional schematics of the solar cell with n-Si/i-a-Si:H/UV-MoO_X_ rear contact. (**b**) *J–V* parameters of solar cells with MoO_X_ and UV-MoO_X_. (**c**) EQE responses and integrated *J*_sc_ values of champion solar cells, with MoO_X_ and UV-MoO_X_ as the HTL. (**d**) Comparison of optical and electrical performance of solar cells in this work with previous works [18,28,33,36,37,38]. (**e**) Free energy loss analysis of solar cells with MoO_X_ and UV-MoO_X_.

Figure 1a illustrates the process of treating MoO_X_ films with UV irradiation, alongside the structure of a n-Si solar cell featuring a full rear contact geometry of UV-MoO_X_/i-a-Si:H/Ag. The cross-sectional TEM image of the rear-side structure of the solar cell is shown in Figure S2. First, we optimized the UV irradiation time and compared the solar cells’ current density–voltage (*J–V*) parameters at different processing times (0 min, 30 min, 60 min, and 120 min) (Figure S3, Supporting Information). When the UV irradiation time was 30 min, the solar cell FF showed a slight improvement. When the UV irradiation time was 60 min, the devices with UV-MoO_X_ film as the HTL performed the best, with the most significant improvement observed in the FF. However, when the irradiation time exceeded 60 min, the FF decreased, leading to a reduction in overall device performance. Figure 1b shows the *J–V* characteristics of n-Si solar cells, using MoO_X_ and UV-MoO_X_ as the HTL. When MoO_X_ is used as the HTL, the efficiency reaches 23.5%. In comparison with MoO_X_-based devices, when UV-MoO_X_ serves as the HTL, *V*_oc_ exhibits a slight decrease; however, the FF is significantly enhanced from 81.62% to 84.82%, leading to an improved device PCE of 24.15%. As shown in Figure 1c, the integrated *J*_sc_ values calculated from the external quantum efficiency (EQE) response of MoO_X_ and UV-MoO_X_ solar cells are 39.60 and 39.76 mA/cm^2^, respectively. EQE curves demonstrate subtle differences in the *J*_sc_ of the UV-MoO_X_ and MoO_X_ devices, but changes in optical performance are not the key factor contributing to improved device performance. Figure 1d presents a comparison of the optical and electrical performance between different solar cells, including this work and previous studies conducted by others. The figure further highlights the substantial improvement in the electrical performance of UV-MoO_X_ devices, relative to MoO_X_. Notably, the UV-MoO_X_ devices in this study exhibit superior PCE in comparison to that of other MoO_X_-based HTL devices featured in previous studies, which can be primarily attributed to the significant improvement in electrical performance. To analyze the reasons for the improvement in the device’s electrical performance, simulations were conducted using quokka 2 under the free energy loss analysis (FELA) method to study the power loss of the devices. As shown in Figure 1e, the power loss is divided into three regions: the front electron-selective contact structure (ESC), the bulk silicon (bulk), and the rear hole-selective contact structure (HSC). Figure 1e also shows that, from the MoO_X_ to the UV-MoO_X_ device, the total power loss at the rear HSC alone decreased from 1.31 to 0.66 mW/cm^2^, while the total power loss in the front ESC and bulk region showed no significant change. The total power loss at the rear HSC was almost equivalent to a reduction in total power loss, which indicates that the improvement in the solar cell’s electrical performance was primarily due to the rear HSC [4]. The contact resistance loss was reduced most significantly, from 1.03 to 0.39 mW/cm^2^, which facilitates excellent contact performance.

**Figure 2:**
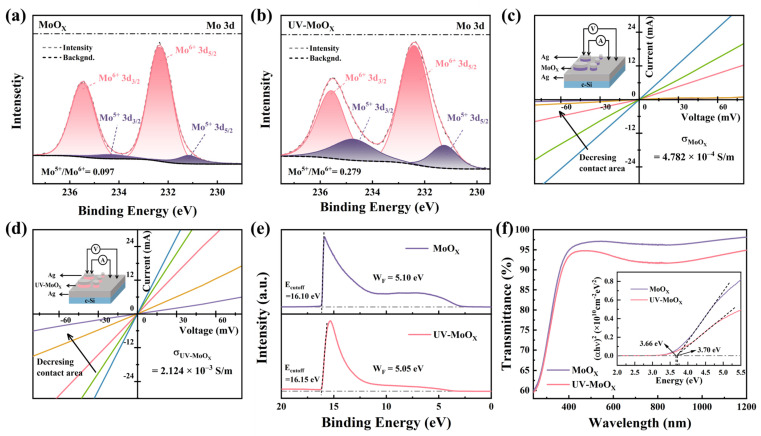
The physical characterization of the MoO_X_ and UV-MoO_X_ films. (**a**,**b**) The Mo 3d XPS spectra of MoO_X_ and UV-MoO_X_. (**c**,**d**) The conductivities of MoO_X_ and UV-MoO_X_ films are extracted by *I–V* curves with different contact area. (**e**) W_F_ of MoO_X_ and UV-MoO_X_ films from UPS spectra. (**f**) UV-vis absorption spectra and transmittance of MoO_X_ and UV-MoO_X_ films. The inset showed the variation in (αhν)^2^ with the photon energy hν.

To evaluate the performance differences between MoO_X_ and UV-MoO_X_ devices, first, the properties of the two films were compared. XPS analysis was employed to investigate the chemical bonding of MoO_X_ and UV-MoO_X_ films deposited onto silicon substrates, as depicted in Figure 2a,b. Both films evaporated by MoO_3_ sources, with a thickness of approximately 7 nm, exhibited Mo^5+^ and Mo^6+^ components in the Mo 3d core level, respectively. The Mo^6+^ 3d_5/2_ and Mo^5+^ 3d_5/2_ peaks were centered at 232.35 and 231.15 eV, respectively, in rigorous agreement with previous works [36,39,40]. In Figure 2a, the Mo^5+^/Mo^6+^ ratio was calculated as 0.097, indicating Mo^6+^ to be the predominant component. In contrast, the Mo^5+^/Mo^6+^ ratio was 0.279 for the UV-MoO_X_ film, as shown in Figure 2b. The different Mo^5+^/Mo^6+^ ratios between the two films indicate that UV irradiation has a reducing effect on MoO_X_ film, which increases Mo^5+^/Mo^6+^ in UV-MoO_X_. Specifically, MoO_X_ generates electron–hole pairs under UV irradiation, according to the following Equation (1):

(1) $$2{Mo}^{6+}O_{3}+2hv\to 2{Mo}^{6+}O_{3}+{2e}^{-}+2h^{+}$$

where h is Planck’s constant; v is the frequency of UV radiation; and e^−^ and h^+^ denote an electron and a hole, respectively. The photo-induced electrons in the conduction band are trapped by Mo^6+^ ions, which are thereby transformed into Mo^5+^ ions [41,42]. Furthermore, the O/Mo ratio of MoO_X_ and UV-MoO_X_ films was determined to be 2.955 and 2.890, respectively (calculated by Figure S3, Supporting Information). The O/Mo ratio of MoO_X_ was similar to that observed in MoO_3_ films [43]. The lower O/Mo ratio in UV-MoO_X_ indicates that there are more oxygen vacancies in MoO_X_ film after UV irradiation. In order to verify the effect of UV irradiation time on the composition of MoO_X_ film, we measured the Mo 3d core level of MoO_X_ film with a 120 min UV treatment (Figure S4, Supporting Information). As UV irradiation time grew, the Mo^5+^/Mo^6+^ ratio rose, while the O/Mo ratio decreased, indicating that increasing the UV irradiation time enhances the concentration of oxygen vacancies. In MoO_X_, oxygen vacancies function as shallow-level donors, boosting carrier concentration and serving as the source of conductivity in the thin films [44,45]. The conductivity of MoO_X_ and UV-MoO_X_ films is shown in Figure 2c,d. The conductivity of MoO_X_ was 4.782 × 10^−4^ S/m, whereas that of UV-MoO_X_ was 2.124 × 10^−3^ S/m, which is almost an order of magnitude higher than that of MoO_X_. The improvement in UV-MoO_X_ conductivity is attributed to the increased carrier concentration induced by oxygen vacancies. The detailed calculation data are shown in Figure S5, Supporting Information. In addition, Figure 2e shows that the W_F_ values of MoO_X_ and UV-MoO_X_ obtained from UPS spectra are 5.10 eV and 5.05 eV, respectively. The W_F_ of UV-MoO_X_ fell by 0.05 eV compared to that of MoO_X_, which was the reason for the decrease in *V*_oc_. Additionally, MoO_X_ treated by UV irradiation for 120 min exhibited a lower W_F_ (Figure S6, Supporting Information). The UV-vis transmittance spectra of MoO_X_ and UV-MoO_X_ films are presented in Figure 2f. UV-MoO_X_ exhibited lower transmittance in the 400–1200 nm range, attributed to free carrier absorption induced by oxygen vacancies [46]. The *E*_g_ of MoO_X_ and UV-MoO_X_ films was determined by Tauc’s equation. The *E_g_* values were found to be 3.70 and 3.66 eV, respectively, as shown in the inset of Figure 2f. UV-MoO_X_ shows almost the same *E_g_* as that of MoO_X_, at only 0.04 eV lower. In addition, we tested the transmittance and absorption of MoO_X_ with different irradiation times. The transmittance of MoO_X_ treated with UV irradiation was lower than that of untreated MoO_X,_ but it remained almost unchanged with different irradiation times. Additionally, there was no significant change in *E_g_* (Figure S6, Supporting Information). In conclusion, the UV-MoO_X_ film exhibits excellent properties when utilized as an HTL, with both high conductivity and an appropriate W_F_.

**Figure 3:**
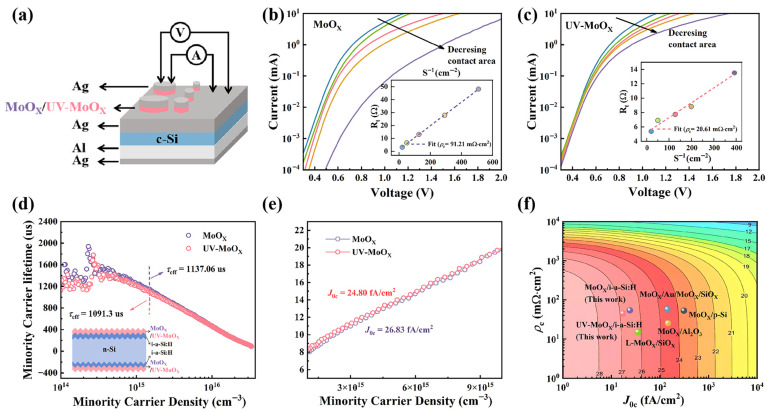
Evaluation of the carrier selectivity of i-a-Si:H/MoO_X_ and i-a-Si:H/UV-MoO_X_ contacts. (**a**) Schematic diagram of the test structure of *ρ*_c_. (**b**,**c**) Experimental measurements of *ρ*_c_ by ECSM for MoO_X_ and UV-MoO_X_ as HTL. The inset showed the total series resistances (R_t_) from different contact area plotted against inverse area (1 S^−1^). (**d**) Injection level-dependent minority carrier lifetime, where the *τ*_eff_ at a Δn of 1.5 × 10^15^ cm^−3^. The inset: the passivated sample with a symmetrical structure for *τ*_eff_ measurement. (**e**) The *J*_0c_ of i-a-Si:H/MoO_X_ and i-a-Si:H/UV-MoO_X_ contacts are extracted by 1/*τ*_corr_ of n-Si passivated by symmetric structure. (**f**) Plot of ideal PCE as a function of contact resistivity *ρ*_c_ and recombination current density *J*_0c_ for different HSCs [33,36,49,50].

In order to evaluate the hole selectivity of the HSCs, the electrical contact performance of n-Si solar cells modified with MoO_X_ and UV-MoO_X_ was measured. The Expanded Cox and Streak method (ECSM) was utilized to extract the *ρ*_c_ of n-Si/i-a-Si:H/MoO_X_ and n-Si/i-a-Si:H/UV-MoO_X_ devices, as shown in Figure 3a. As shown in Figure 3b,c, the colored lines are the dark *I–V* curves of the ECSM disks with different contact areas. The inset in Figure 3b,c shows the total series resistance (*R*_t_) from different disks, plotted against an inverse area (S^−1^). The *ρ*_c_ value of n-Si/i-a-Si:H/UV-MoO_X_ (20.61 mΩ·cm^2^) demonstrated a significant reduction compared to n-Si/i-a-Si:H/MoO_X_ (91.21 mΩ·cm^2^). UV-MoO_X_ produced more oxygen vacancies, which increased the carrier concentration and achieved better contact performance. The low *ρ*_c_ values of devices using UV-MoO_X_ as the HTL are highly recommended for achieving high FF and PCE for *c*-Si solar cells. A symmetric structure, shown in the inset of Figure 3d, was used to characterize the minority carrier lifetime (*τ*_eff_) for the HTL, based on MoO_X_ and UV-MoO_X_ using the quasi-steady-state-photoconductance (QSSPC) method. At an injection level of 1.5 × 10^15^ cm^−3^, the *τ*_eff_ value of the MoO_X_-based device was 1137.06 μs. Replacing the MoO_X_ layer with a UV-MoO_X_ layer results in a similar *τ*_eff_ of 1091.3 μs, indicating that MoO_X_ and UV-MoO_X_ possess comparable field passivation ability. It is further demonstrated that a slight decrease in the W_F_ hardly weakens the field passivation effect of the UV-MoO_X_ layer. Meanwhile, the *J*_0c_ was extracted from the *τ*_eff_ measurement, representing the flux of non-collected charge carriers to the contact. It is noteworthy that the wafer passivated solely with the i-a-Si:H layer also exhibiting excellent surface passivation performance, with an effective carrier lifetime reaching 1.12 ms (Figure S7): a level comparable to that observed in devices with deposited MoO_X_ or UV-MoO_X_. This result confirms that the prepared 8 nm-thick i-a-Si:H film provides high-quality interface passivation. Furthermore, it indicates that subsequent processing steps, including deposition and ultraviolet treatment, introduced almost no adverse effects on the initial passivation quality. Figure 3e shows the Auger-corrected inverse effective lifetime (1/*τ*_corr_) as a function of minority carrier concentration, where 1/*τ*_corr_ = 1/*τ*_eff_ − 1/*τ*_Auger_ [47]. The specific calculation process is described in detail in Section 2. The *J*_0c_ value of the n-Si/i-a-Si:H/MoO_X_ contact was approximately 24.80 fA/cm^2^, while n-Si/i-a-Si:H/UV-MoO_X_ has a *J*_0c_ value of 26.83 fA/cm^2^. The results show that the presence of more defects (vacancy oxygen) in UV-MoO_X_ contacts leads to an increase in carrier recombination and *J*_0c_. In summary, although the replacement of the MoO_X_ layer with a UV-MoO_X_ layer leads to a slight increase in the *J*_0c_, it greatly reduces the *ρ*_c_, which improves the carrier selectivity. In addition, a low *ρ*_c_ most likely suggests a high FF [48]. Figure 3f plots the ideal PCE as a function of *ρ*_c_ and *J*_0c_ for MoO_X_ and UV-MoO_X_ contacts, as well as the *ρ*_c_ and *J*_0c_ values from previous works. First, compared with other passivation layers (SiO_X_ or Al_2_O_3_), i-a-Si:H exhibits a better passivation effect, reducing carrier recombination, and thus resulting in a smaller *J*_0c_. Furthermore, UV-MoO_X_ achieves a lower *ρ*_c_ while maintaining a small *J*_0c_. Due to its excellent carrier selectivity, UV-MoO_X_ has as great potential as an HTL.

**Figure 4:**
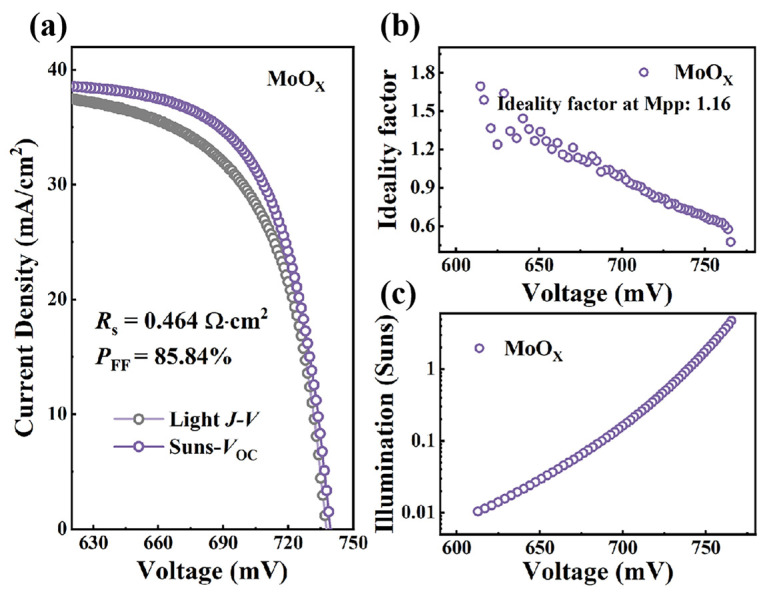
Electrical performance of solar cells with MoO_X_ as HTL. (**a**) Experimental *J–V* curves and the Suns-*V*_oc_ curves for the MoO_X_ cell. The pseudo-*J–V* curves are constructed by a *V*_oc_-*J*_sc_ (Suns) plot at different intensities. The illumination intensity is monitored by a calibrated reference cell. (**b**) Ideality factor calculated from the Suns-*V*_oc_ curve for the solar cell with MoO_X_. (**c**) Illumination–voltage curve of solar cells with MoO_X_.

**Figure 5:**
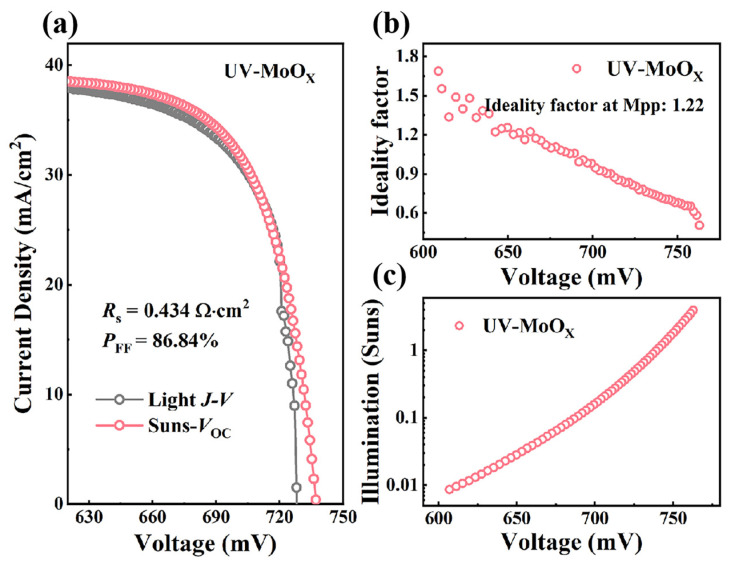
Electrical performance of solar cells with UV-MoO_X_ as HTL. (**a**) Experimental *J–V* curves and the Suns-*V*_oc_ curves for the UV-MoO_X_ cell. The pseudo-*J–V* curves are constructed by a *V*_oc_-*J*_sc_ (Suns) plot at different intensities. The illumination intensity is monitored by a calibrated reference cell. (**b**) Ideality factor calculated from the Suns-*V*_oc_ curve for the solar cell with UV-MoO_X_. (**c**) Illumination–voltage curve of solar cells with UV-MoO_X_.

To explore the main contribution to the improvement in FF, Suns-*V*_oc_ measurements of solar cells with MoO_X_ and UV-MoO_X_ were performed. The Suns-*V*_oc_ pseudo-*J–V* curve, constructed from *V*_oc_ measurements at different incident light intensities (Suns), is unaffected by the voltage drop due to series resistance (*R*_s_) [51]. The experimental curves include the real *J–V* curve (purple-colored curve) and the pseudo-*J–V* curve obtained by a Suns-*V*_oc_ measurement (pink-colored curve). By comparing the real *J–V* curves and pseudo-*J–V* curves in Figure 4a and Figure 5a, the device with UV-MoO_X_ demonstrates a significantly reduced *R*_s_ of 0.434 Ω·cm^2^ compared to the MoO_X_-based device, with a value of 0.464 Ω·cm^2^, indicating that the UV-MoO_X_ device exhibits lower resistance loss. Pseudo-FF (pFF: FF excluding *R*_s_ power loss) values of 85.84% and 86.84% are extracted from the pseudo-*J–V* curves of MoO_X_ and UV-MoO_X_ solar cells. Compared with the FF, the pFF of MoO_X_ and UV-MoO_X_ solar cells increase by 4.22% and 2.02%, respectively, which also indicates the variation trend of *R*_s_. Next, to evaluate the impact of additional recombination pathways in the different cells, the ideality factor was calculated from the Suns-*V*_oc_ data. The ideality factors of MoO_X_ and UV-MoO_X_ devices from the Suns-*V*_oc_ measurement are shown in Figure 4b and Figure 5b. Typically, the ideality factor ranges from one to two for real devices. A value of *n* > 1 indicates that traps are involved in the carrier recombination mechanism in solar cells [52,53]. In contrast, when the value of *n* < 1, it may be attributed to the enhanced Auger recombination mechanism [54]. Thus, the ideality factor reflects the impact of traps on carrier recombination within photovoltaic devices. The ideality factor around the MPP (V = 664 mV) for the MoO_X_ cells is approximately 1.16. Correspondingly, the ideality factor of the UV-MoO_X_ device at the MPP (V = 660 mV) is similar to that of the MoO_X_ device, with a value of 1.22. The slight difference in the ideality factor at the MPP indicates that the UV-MoO_X_ device exhibits more carrier recombination; however, the discrepancy is negligible, which is consistent with the previous analysis of *J*_0c_. In addition, Figure 4c and Figure 5c show a transition from a low to high injection level, as the voltage shifts from the low-voltage to the high-voltage region. Both the MoO_X_ and UV-MoO_X_ devices exhibit a decrease in the ideality factor in the high-voltage region. This is due to the increasing role of Auger recombination in the high-voltage region.

To further validate the reliability of our results, we have now supplemented the study with additional data from devices subjected to 180 °C thermal annealing (see Figure S8). Minority carrier lifetime test samples with a MoO_X_/i-a-Si:H/n-Si/i-a-Si:H/MoO_X_ symmetric structure were prepared and annealed at 180 °C for 10 min in a nitrogen atmosphere. The measured minority carrier lifetimes were 1091.3 μs for the as-processed sample and 1148.42 μs for the annealed sample, revealing nearly identical performance between the two. This result confirms that the UV treatment employed in this work caused only minimal damage to the Si–H_X_ bonds.

## 4. Conclusions

In summary, we demonstrated that UV irradiation enables the modulation of oxygen vacancy density in MoO_X_ films to improve the electrical performance of *c*-Si solar cells. At a UV treatment time of 60 min, the MoO_X_ film exhibits both a relatively high work function of 5.05 eV and high conductivity of 2.124 × 10^−3^ S/m, which effectively balances the influence of film properties on *V*_oc_ and FF. Additionally, UV-MoO_X_ films still have a comparable *τ*_eff_ to MoO_X_ films, indicating that UV-MoO_X_ provides a field passivation effect that is similar to that of MoO_X_. The key improvement lies in the significant reduction in the *ρ*_c_ of UV-MoO_X_ compared to MoO_X_. A *ρ*_c_ of 20.61 mΩ·cm^2^ indicates that UV-MoO_X_ films possess superior contact characteristics, facilitating the selective transport of carriers. Further analysis comparing the *J–V* curve and the pseudo-light-*J–V* curve from Suns-*V*_oc_ measurements demonstrates that UV-MoO_X_ has a smaller *R*_s_ of 0.434 Ω·cm^2^ and an excellent pFF of 86.84%. These advantages contribute to the enhanced performance of solar cells employing UV-MoO_X_ as the HTL, as evidenced by the state-of-the-art PCE of 24.15%. This work lays the foundation for attaining the highest PCE in *c*-Si solar cells using UV-MoO_X_ as the HTLs. Furthermore, the successful application of UV-MoO_X_ in *c*-Si solar cells provides valuable new insights into the controllable modulation of properties in high-W_F_ TMOs.

## Data Availability

The original contributions presented in this study are included in the article and Supplementary Materials. Further inquiries can be directed to the corresponding author.

## Supplementary Materials

The following supporting information can be downloaded at: https://www.mdpi.com/article/10.3390/ma18225167/s1. Figure S1: Fabrication flowchart of a crystalline silicon solar cell with UV-MoO_X_ HTL; Figure S2: Cross-sectional AC-TEM images of the n-Si/a-Si:H(i)/UV-MoO_X_/Ag, i-a-Si:H ~8 nm, MoOx ~7 nm, Ag ~200 nm; Figure S3: *J*-*V* parameters of devices with different UV irradiation time (0 min, 30 min, 60 min and 120 min); Figure S4: (a) The XPS spectra of MoO_X_ film with different UV irradiation time (0 min, 60 min and 120 min), Where MoO_X_ and UV-MoO_X_ represent 0 min and 60 min, respectively. (b) The Mo 3d XPS spectra of MoO_X_ film with 120 min UV irradiation. (c) O/Mo ratios of MoO_X_ film with different UV irradiation time (0 min, 60 min and 120 min); Figure S5: Plots of total resistance (Rt) versus 1/S (fitted by dashed curves) of (a) p-Si/Ag/MoO_X_/Ag and b) p-Si/Ag/UV-MoO_X_/Ag structures; Figure S6: (a) UPS spectra of MoO_X_ film with 120 min UV irradiation. (b) UV-vis absorption spectra and transmittance of MoO_X_ film with 120 min UV irradiation. The inset showed the variation of (αhν)^2^ with the photon energy hν; Figure S7: Injection level-dependent minority carrier lifetime with i-a-Si:H/n-Si/i-a-Si:H structures, where the *τ*_eff_ at a Δn of 1.5 × 10^15^ cm^−3^; Figure S8: Injection level-dependent minority carrier lifetime with annealed UV-MoO_X_/i-a-Si:H/n-Si/i-a-Si:H/UV-MoO_X_ structures, where the *τ*_eff_ at a Δn of 1.5 × 10^15^ cm^−3^; Table S1: *J-V* parameters of solar cells fabricated with continuous deposition and interrupted deposition.; Table S2: Input parameters of Quokka2 simulation for MoO_X_ cell and UV-MoO_X_ cell. Note S1: Expanded Cox-Strack Method. References [26,55,56,57] are cited in the Supplementary Materials.
