# Supplementary material for: UV-Engineered Oxygen Vacancies in MoOX Interlayers Enable 24.15% Efficiency for Crystalline Silicon Solar Cells
Source: Materials (Basel). 2025 Nov 13;18(22):5167. doi: 10.3390/ma18225167 (PMC12654205; doi:10.3390/ma18225167)
Supplement: Supplementary file 1 [file materials-18-05167-s001.zip › materials-3961637-supplementary.pdf]

Supporting Information

UV-Engineered Oxygen Vacancies in MoO_x Interlayers Enable 24.15% Efficiency for Crystalline Silicon Solar Cells

Linfeng Yang [1,†], Wanyu Lu [1,†], Jingjie Li [2], Shaopeng Chen [1], Tinghao Liu [3], Dayong Yuan [1], Yin Wang [1], Ji Zhu [1], Hui Yan [3], Yongzhe Zhang [1] and Qian Kang [1,*]

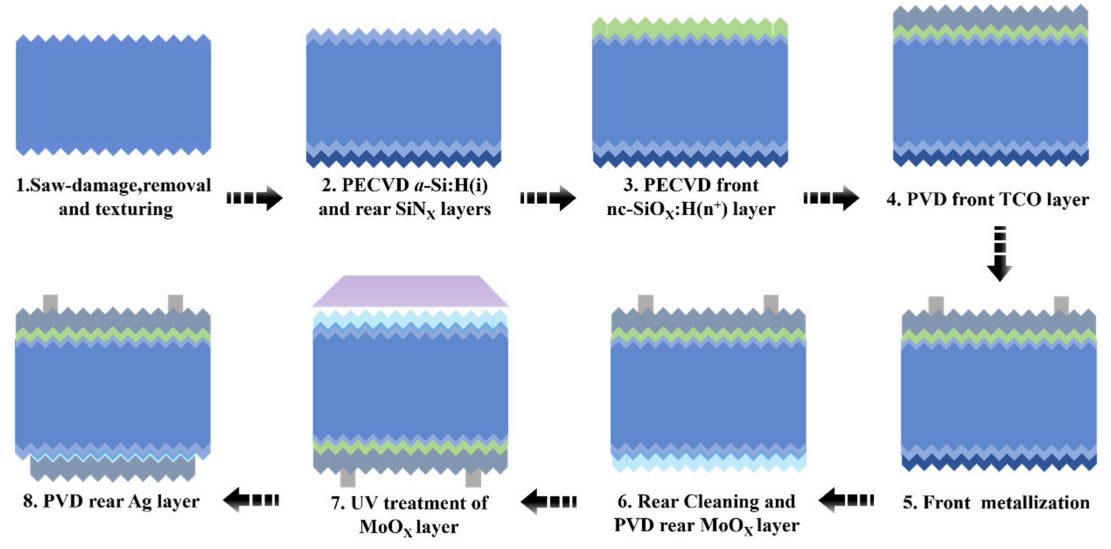

Figure S1. Fabrication flowchart of a crystalline silicon solar cell with UV-MoO_x HTL.

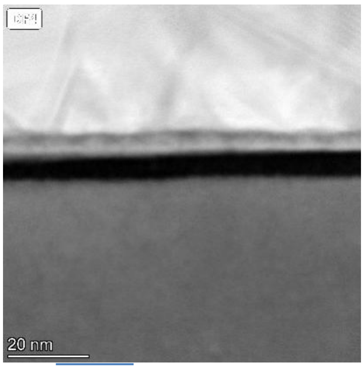

Figure S2. Cross-sectional AC-TEM images of the n-Si/a-Si:H(i)/UV-MoO_x/Ag, i-a-Si:H ~8 nm, MoOx ~7 nm, Ag ~200 nm

Table S1. *J-V* parameters of solar cells fabricated with continuous deposition and interrupted deposition.

	J_{SC} (mA/cm^2)	V_{OC} (mV)	FF (%)	PCE (%)
continuous deposition	38.84	721	81.47	22.84
nterrupted deposition	38.77	722	81.35	22.78

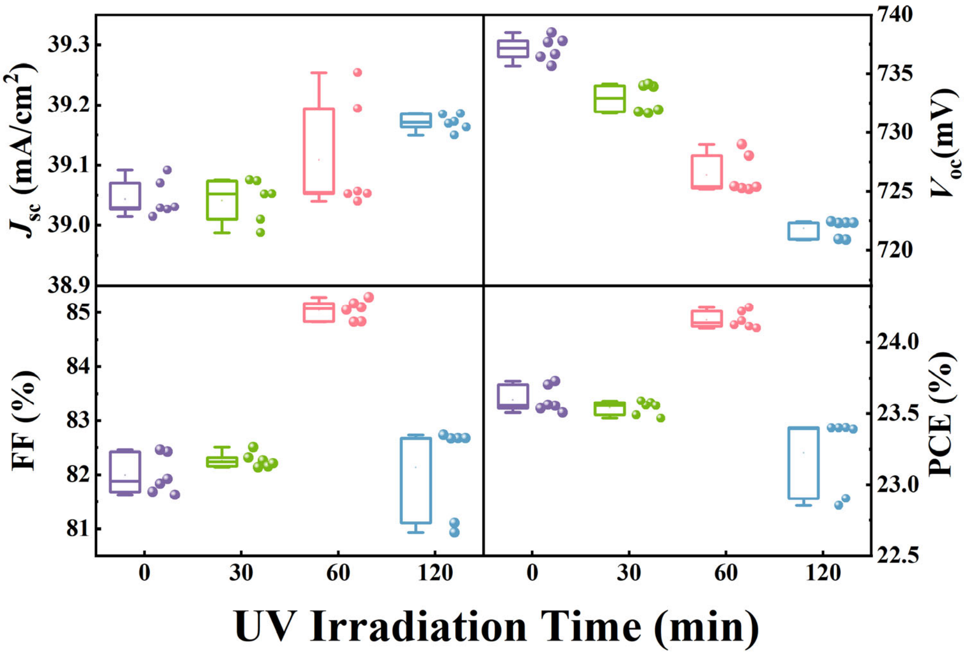

Figure S3. *J-V* parameters of devices with different UV irradiation time (0 min, 30 min, 60 min and 120 min)

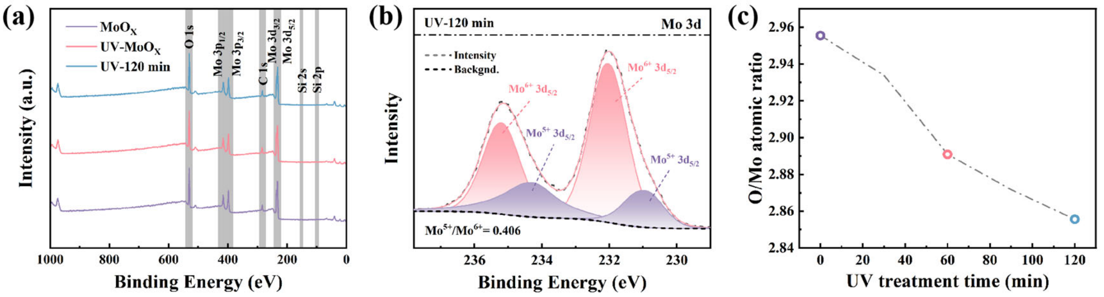

Figure S4. (a) The XPS spectra of MoO_X film with different UV irradiation time (0 min, 60 min and 120 min), Where MoO_X and UV-MoO_X represent 0 min and 60 min, respectively. (b) The Mo 3d XPS spectra of MoO_X film with 120 min UV irradiation. (c) O/Mo ratios of MoO_X film with different UV irradiation time (0 min, 60 min and 120 min).

The presence of Si 2s and Si 2p peaks in the full spectrum indicates the presence of non-stoichiometric SiO_X, therefore the O/Mo ratio cannot be calculated from the full spectrum. In this paper, we calculated the O/Mo ratio according to the reference [26]. The formula for calculating O/Mo ratio is as follows: $\eta_{O/Mo} = (\eta_{Mo6+} \times 3 + \eta_{Mo5+} \times 2.5) / (\eta_{Mo6+} + \eta_{Mo5+})$.

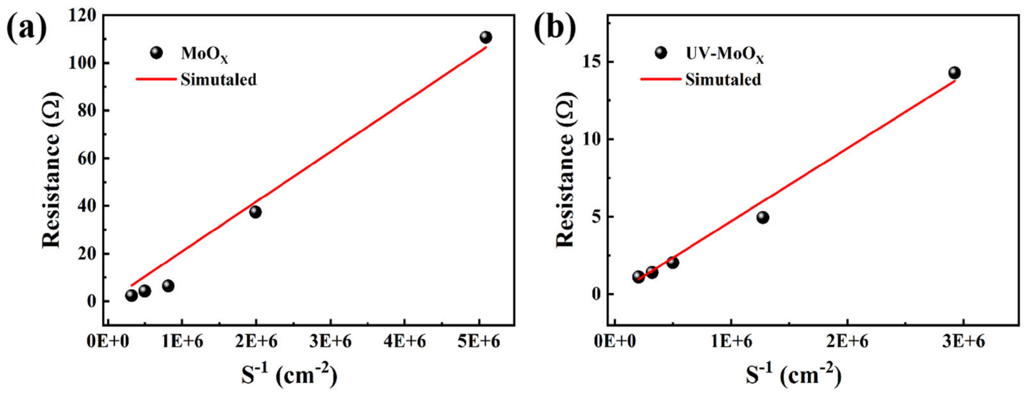

Figure S5. Plots of total resistance (R_t) versus 1/S (fitted by dashed curves) of (a) p-Si/Ag/MoO_x/Ag and (b) p-Si/Ag/UV-MoO_x/Ag structures.

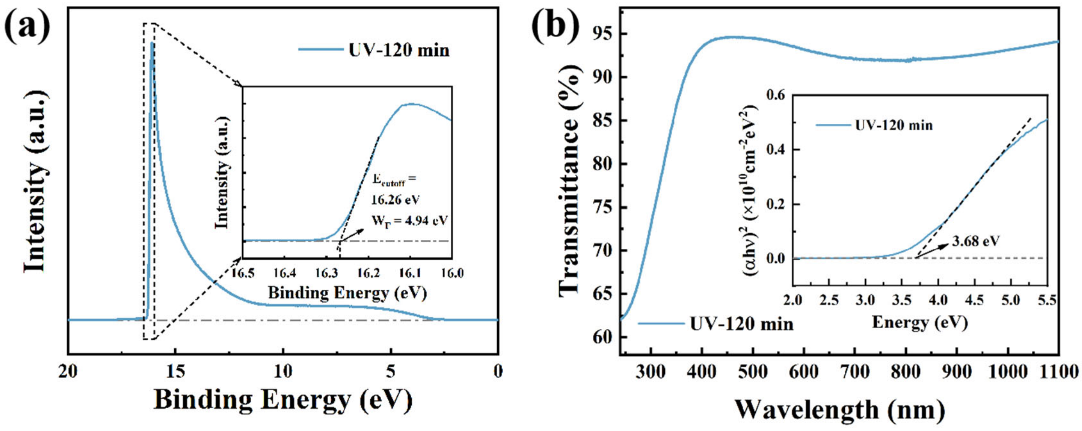

Figure S6. (a) UPS spectra of MoO_x film with 120 min UV irradiation. (b) UV-vis absorption spectra and transmittance of MoO_x film with 120 min UV irradiation. The inset showed the variation of $(\alpha h\nu)^2$ with the photon energy $h\nu$.

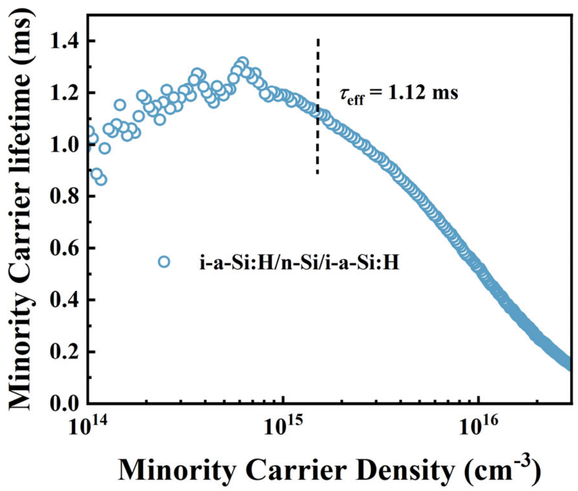

Figure S7. Injection level-dependent minority carrier lifetime with i-a-Si:H/n-Si/i-a-Si:H structures, where the τ_{eff} at a Δn of 1.5×10^{15} cm^{-3}.

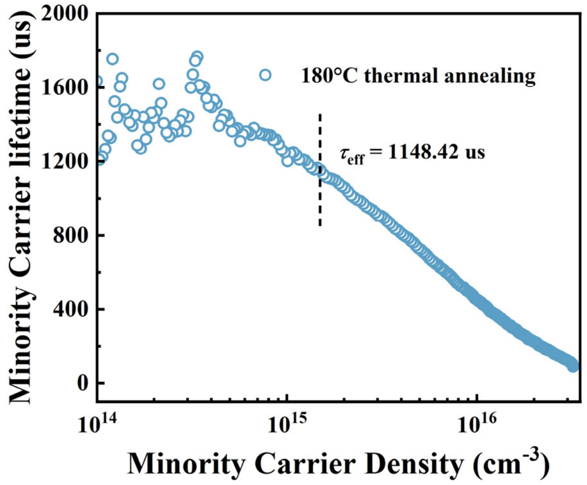

Figure S8. Injection level-dependent minority carrier lifetime with annealed UV-MoO_x/i-a-Si:H/n-Si/i-a-Si:H/UV-MoO_x structures, where the τ_{eff} at a Δn of 1.5×10^{15} cm^{-3}.

Note S1 Expanded Cox-Strack Method

The expanded Cox-Strack Method (ECSM) is proposed to precisely extract the ρ_c of MoO_X/n-Si heterojunction, achieving a generally lower coefficient of variation [55, 56]. The current-voltage (*I–V*) is measured from the circular contact through the substrate to its grounded metalized backside. The resistance calculated by the *I–V* line is the total resistance R_T, including contact resistance R_c (between metal and semiconductor), spreading resistance R_S (the semiconductor), and residual resistance R_0 (due to the substrate or the backside contact).

$$R_T = R_C + R_S + R_0$$

The contact resistance R_C is related to the top electrode diameter, which is defined as:

$$R_C = \frac{\rho_c}{\pi d^2/4}$$

where d is the diameter of the circular electrode and ρ_c is the specific contact resistance. The value of R_T is obtained using Cheung's method [57] and then brought into the traditional CSM to extract ρ_c. The equation for obtaining R_T is

$$\frac{\mathrm{dV}}{\mathrm{dln(I)}} = R_T I + \frac{nkT}{q}$$

The spreading resistance R_S can be proved to be negligible, so that $R_T \approx R_C + R_0$. So, the Expanded Cox-Strack equation is

$$R_T = \frac{\rho_c}{\pi d^2/4} + R_0$$

Therefore, the Expanded Cox-Strack contact resistivity determination procedure consists of measuring R_T on a range of top contacts with different diameters 1/d and determining ϱ_c from the R_T dependence.

Table S2. Input parameters of Quokka2 simulation for MoO_X cell and UV-MoO_X cell.

	MoO_X	UV-MoO_X
**Unit cell dimension**		
Front width (um)	1000	1000
Rear width (um)	1000	1000
Thickness (um)	130	130
**Bulk**		
Doping type	n-type	n-type
Resistivity (Ω·cm)	1.5	1.5
τ_{SRH} (ms)	40	40
B_{rad} (cm^3/s) *	1.89×10^{-15}	1.89×10^{-15}
Auger mode	Richter 2012	Richter 2012
**Front surface**		
Sheet resistance (Ω/□)	150	150
J_{01} (fA/cm^2)	1	1
Contact half width (μm)	20	20
**Rear surface**		
Sheet resistance (Ω/□)	200	150
J_{01} (fA/cm^2)	24.8	26.83

Contact half width (μm)	80	80
**Generation**		
Shading width (μm)	80	80
Optical pathlength factor (Z)	$4n^2$	$4n^2$
Transmission	0.9	0.9

26. Li, J.; Pan, T.; Wang, J.; Cao, S.; Lin, Y.; Hoex, B.; Ma, Z.; Lu, L.; Yang, L.; Sun, B.; Li, D., Bilayer MoOX/CrOX Passivating Contact Targeting Highly Stable Silicon Heterojunction Solar Cells. *ACS Applied Materials & Interfaces* **2020,** 12, (32), 36778-36786.

55. Chen, L.; Lin, H.; Liu, Z.; Wu, T.; Pang, Y.; Gao, P.; Shen, W., Realization of a General Method for Extracting Specific Contact Resistance of Silicon-Based Dopant-Free Heterojunctions. *Solar RRL* **2021,** 6, (2), 2100394.

56. Wang, W.; Lin, H.; Yang, Z.; Wang, Z.; Wang, J.; Zhang, L.; Liao, M.; Zeng, Y.; Gao, P.; Yan, B.; Ye, J., An Expanded Cox and Strack Method for Precise Extraction of Specific Contact Resistance of Transition Metal Oxide/n-Silicon Heterojunction. *IEEE Journal of Photovoltaics* **2019,** 9, (4), 1113-1120.

57. Cheung, S. K.; Cheung, N. W., Extraction of Schottky diode parameters from forward current-voltage characteristics. *Applied Physics Letters* **1986,** 49, (2), 85-87.
